# Voluntary Exercise Prevents Lead-Induced Elevation of Oxidative Stress and Inflammation Markers in Male Rat Blood

**DOI:** 10.1155/2013/320704

**Published:** 2013-10-03

**Authors:** Mustafa Mohammadi, Rana Ghaznavi, Rana Keyhanmanesh, Hamid Reza Sadeghipour, Roya Naderi, Hossein Mohammadi

**Affiliations:** ^1^Drug Applied Research Center, Tabriz University of Medical Sciences, Tabriz 51656-65811, Iran; ^2^Department of Physiology, Faculty of Medicine, Tehran University of Medical Sciences, Tehran, Iran; ^3^Dental School, Tabriz University of Medical Sciences, Tabriz, Iran

## Abstract

Regular mild exercise enhances antioxidant and anti-inflammatory systems of the body. The present study investigates voluntary exercise effects on lead toxicity as a known oxidative stressor. Male Sprague-Dawley rats were randomly divided into 2 groups. Sedentary control: the animals were housed 7 weeks in the regular cages. Exercise group: the animals were housed 7 weeks in the running wheel equipped cages, that is, the animal model of voluntary exercise. During the 7th week, all animals were administered lead acetate. Blood samples were collected at the end of the 6th week and 7th week (before and after lead administrations). Glutathione peroxidase (GPx), superoxide dismutase (SOD), catalase (CAT), malondialdehyde (MDA), and tumor necrosis factor (TNF-*α*) were measured in the samples. Our results showed that lead administration reduced blood SOD, GPx and CAT and increased TNF-*α*; in the controls, but in the exercise group, changes were not statistically significant. MDA in both groups increased after lead injections but it was significantly lower in exercise group compared to the sedentary animals. We concluded that voluntary exercise may be considered as a preventive tool against lead-induced oxidative stress and inflammation.

## 1. Introduction

Regular physical exercise has known health benefits. Exercise decreases the risk of cardiovascular diseases, cancer, and diabetes. Different mechanisms have been demonstrated for exercise beneficial effects, including upregulation of anti-inflammatory and antioxidant pathways [1]. 

Oxidative stress is an imbalance between the free radical production and antioxidant defense systems of the body and has been implicated in many diseases [2]. Exercise beneficial effects have been studied in different cases of the increased oxidative stress including diabetes [3], aging [4], and hypercholesterolemia [5]. 

Although many beneficial effects have been reported for mild/moderate exercise, exhaustive exercise induces oxidative stress through production of reactive oxygen species and can cause damage to muscle tissue and other organs [6, 7]. In the animal model of voluntary exercise, the animal has free access to a running wheel and uses the wheel according its physiological threshold for physical activity. So, voluntary exercise is regarded as mild/moderate exercise [8].

Lead is a heavy metal with wide toxic effects on brain, heart, liver, and kidney. Although lead toxicity has been relatively controlled in industries, it is still an important health issue in many countries. Local surveillance efforts in Iran prove that lead continues to be found often at toxic levels in the air, soil, and food supply [9]. Lead was reported as the most toxic metal in fresh water of Malaysia [10]. Consumption of vegetables produced on lead-contaminated soils is a health risk in Nigeria [11]. It is also the most common environmental toxicity in the United States of America [9]. Oxidative stress may play the main role in toxicity of lead due to imbalance in oxidant-anti oxidant homeostasis [12]. 

As mentioned above, the effects of exercise have been deliberated in some oxidative stress conditions, but in the case of lead-induced oxidative stress, it is remained to be studied. The aim of the present study is investigation of the voluntary exercise effects as an antioxidant and anti-inflammatory systems enhancer on body resistance against lead-induced oxidative stress and inflammation.

## 2. Materials and Methods

### 2.1. Animals

20 Male Sprague-Dawley rats weighing 250 ± 10 g were housed under controlled environmental conditions (24 ± 2°C and 12 h light-dark cycle) and allowed free access to standard rat chow and tap water. Animal care was in compliance with the guidelines of the Animal and Human Ethical Committee of Tabriz Medical Sciences University.

### 2.2. Experiments Protocol

 Animals were randomly divided into 2 groups (10 rats in each): sedentary control: the animals were housed 7 weeks in the regular cages. Exercise group: the animals were housed 7 weeks in the running wheel equipped cages (Tajhiz-Gostar, Tehran, Iran), that is, animal model of the voluntary exercise [8]. The wheels were attached to a permanent sensor that activated a digital counter of wheel revolutions. Total wheel revolutions were recorded daily, with total distance run per day determined by multiplying the number of wheel rotations by wheel circumference. During the 7th week all animals were administered lead acetate (15 mg/kg body weight, ip, 7 days) [13]. A blood sample was collected at the end of the 6th week before lead administration. After the experiments the rats were anaesthetized with ketamine (44 mg/kg, ip) and chlorpromazine (30 mg/kg, ip) [14] and blood samples were collected. Then the animals were sacrificed under deep anesthesia by heart dissections. A portion of the each sample was stored as whole blood in −70°C for Glutathione peroxidase (GPx), superoxide dismutase (SOD) and catalase (CAT) activities and malondialdehyde (MDA) measurements. Plasma of the rest of each sample was extracted and stored at −70°C for further tumor necrosis factor (TNF-*α*) assay.

### 2.3. Measurement of Antioxidant Enzymes Activities

Whole blood samples were used for determination of GPx, SOD and CAT. SOD activity was assayed by commercial kit (RANSOD, Randox co., Antrim, United Kingdom) according to Delmas-Beauvieux et al. method [15]. This method employs xanthine and xanthine oxidase to generate superoxide radicals which react with 2-(4-iodophenyl)-3-(4-nitrophenol)-5-phenyltetrazolium chloride to form a red formazan dye. The superoxide dismutase activity is then measured by the degree of inhibition of this reaction and was expressed as U/g hemoglobin (Hb). 

Glutathione peroxidase (GPx) activity was determined using commercial kit (RANSEL, Randox co., Antrim, United Kingdom) according to the method of Paglia and Valentine [16]. Briefly, in the presence of glutathione reductase and NADPH, oxidized glutathione is immediately converted to the reduced form with a concomitant oxidation of NADPH to NAD+. The decrease in absorbance at 340 nm (37°C) was measured. GPx concentration was calculated by the related formula and expressed as U/g Hb.

Catalase activity was measured using the Aebi method [17]. Briefly, measurement was performed based on dissociation rate of H_2_O_2_ in 240 nm at 20°C. Whole blood lysates were centrifuged. The adequate amount of supernatant was added to a reaction mixture that contained 0.002% Triton X-100, 0.1 mM EDTA, 0.5 M potassium phosphate buffer, and 15 mM H_2_O_2_ in 1 mL final volume at pH 7.0. Activity was calculated as the decomposition rate within the initial 15 s and expressed as K/g Hb.

### 2.4. Lipid Peroxidation Study

MDA as the end-product of lipid peroxidation was measured in the blood samples according to the Esterbauer and Cheeseman method [18]. MDA reacts with thiobarbituric acid and produces a pink pigment that has a maximum absorption at 532 nm.

### 2.5. Assay of Inflammatory Cytokine

The concentration of TNF-*α* as inflammation marker was determined by an enzyme-linked immunosorbent assay (ELISA) in 450 nm wave length using commercial rat TNF-*α* assay kit (eBioscience, San Diego, CA, USA). The assays were carried out according to the manufacturers' instructions.

### 2.6. Statistics

All numerical data are expressed as the mean ± SEM. SOD, GPx, CAT and TNF-*α* were analyzed using paired sample *t*-test. A *P* value less than 0.05 was considered statistically significant.

## 3. Results

Rats with free access to running wheels ran a total of 1453 ± 170 m per day.

### 3.1. Antioxidant Enzymes

**Figure 1:**
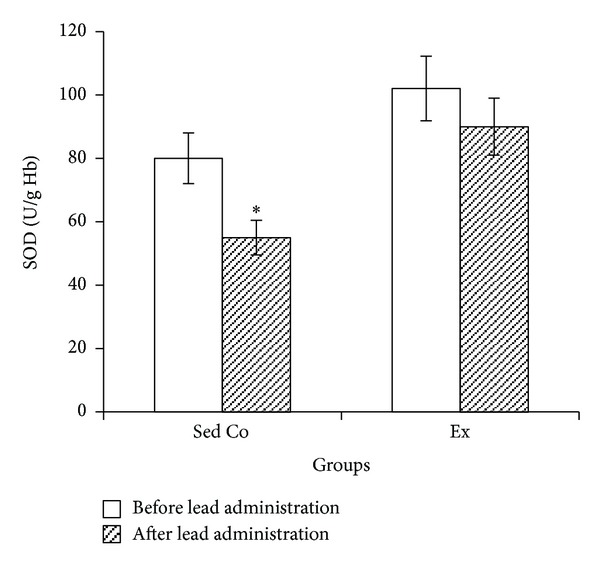
Blood superoxide dismutase in sedentary control and exercise groups before and after lead administrations. Seven days of lead injections (15 mg/Kg body weight, ip) to sedentary animals housed in the regular cages caused significant reduction of blood SOD (*P < 0.05 compared to the “before lead administration”); in Ex animals housed in the wheel equipped cages, SOD changes after the same dose lead administration were not significant. The data are presented as means ± S.E.M. SOD: superoxide dismutase; Sed Co: sedentary control group; Ex: exercise group.

**Figure 2:**
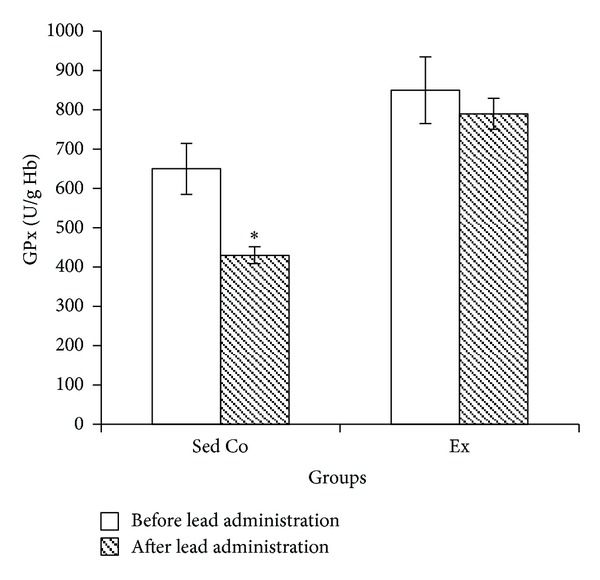
Blood glutathione peroxidase in sedentary control and exercise groups before and after lead administrations. Seven days of lead injections (15 mg/Kg body weight, ip) to sedentary animals housed in the regular cages caused significant reduction of blood GPx (*P < 0.05 compared to the “before lead administration”); in Ex animals housed in the wheel equipped cages, GPx changes after the same dose lead administrations were not significant. The data are presented as means ± S.E.M. GPx: glutathione peroxidase; Sed Co: sedentary control group; Ex: exercise group.

**Figure 3:**
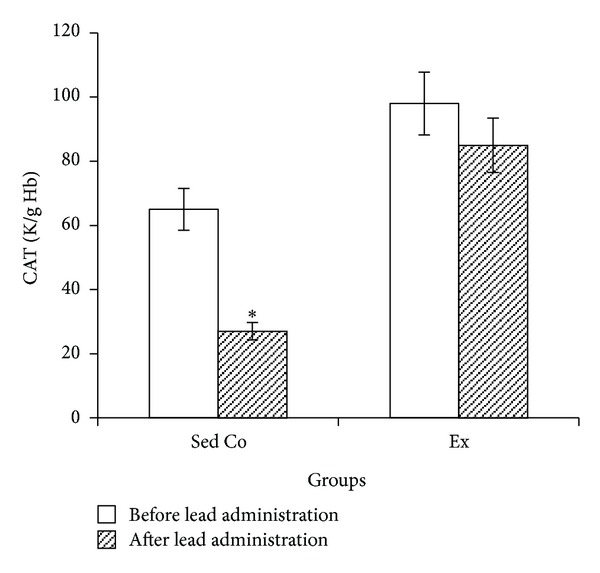
Blood catalase activity in sedentary control and exercise groups before and after lead administrations. Seven days of lead injections (15 mg/Kg body weight, ip) to sedentary animals housed in the regular cages caused significant reduction of blood CAT (*P < 0.05 compared to the “before lead administration”); in Ex animals housed in the wheel equipped cages, CAT changes after the same dose lead administrations were not significant. The data are presented as means ± S.E.M. CAT: catalase; Sed Co: sedentary control group; Ex: exercise group.

Lead administration in sedentary control group caused significant reduction in blood SOD, GPx, and CAT activities while in exercise group lead-induced SOD, GPx, and CAT changes were not significant (Figures 1, 2, and 3, resp.).

### 3.2. Lipid Peroxidation Study

**Figure 4:**
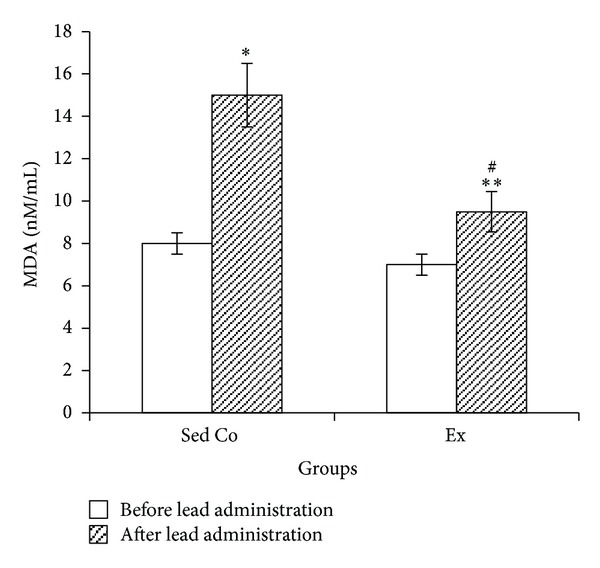
Blood malondialdehyde in sedentary control and exercise groups before and after lead administrations. Seven days of lead injections (15 mg/kg body weight, ip) to the both sedentary and exercising animals caused significant raise of blood MDA (*P < 0.05 compared to the “before lead administration” in Sed Co group, **P < 0.05 compared to the “before lead administration” in Ex group); in Ex animals MDA levels after the lead administrations were significantly lower than the Sed Co lead-injected animals (^#^ *P* < 0.05 compared to the “after lead administration” in the Sed Co group). The data are presented as means ± S.E.M. MDA: malondialdehyde; Sed Co: sedentary control group; Ex: exercise group.

Lead administration in the both groups significantly increased blood MDA. Lead-treated animals of Ex group had lower MDA levels when compared to the lead-treated animals of Sed Co group (Figure 4). 

### 3.3. Inflammatory Cytokine

**Figure 5:**
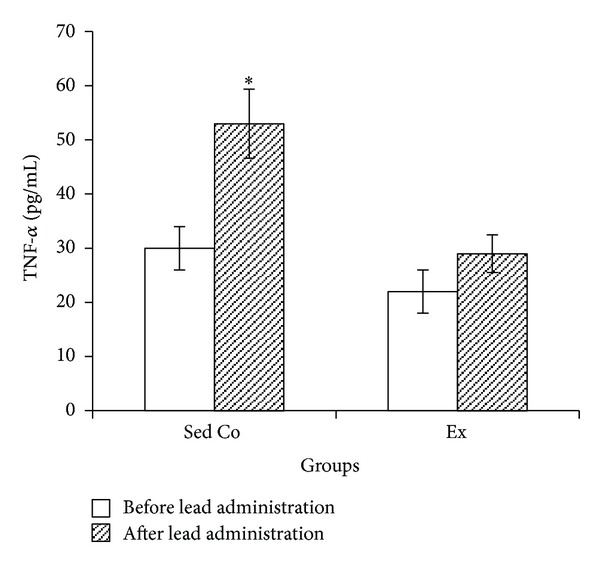
Plasma tumor necrosis factor-*α* in sedentary control and exercise groups before and after lead administrations. Seven days of lead injections (15 mg/Kg body weight, ip) to sedentary animals housed in the regular cages caused significant increase of plasma TNF-*α* (*P < 0.05 compared to the “before lead administration”); in Ex animals housed in the wheel equipped cages, plasma TNF-*α* changes after the same dose lead administrations were not significant. The data are presented as means ± S.E.M. TNF-*α*: tumor necrosis factor-*α*; Sed Co: sedentary control group; Ex: exercise group.

Mean plasma TNF-*α* level was significantly increased after 7 days of lead injections in sedentary control group but in exercise group TNF-*α* did not significantly change after lead administration (Figure 5). 

## 4. Discussion

According to the results of the present study lead administration induced oxidative stress (decreased blood SOD, GPx, and CAT and increased MDA) and inflammation (increased TNF-*α*). 7 weeks voluntary exercise prevented lead-induced oxidative stress and inflammation. In the case of lipid peroxidation, exercise could not completely prevent the lead toxicity but limited it to a lower extent.

Our results are in accordance with the findings of the previous studies that have suggested protective role for exercise against oxidative and inflammatory stresses. Golbidi et al. have shown that exercise improves diabetic patients' health condition by its anti-inflammatory and antioxidant effects [3]. Another study has reported prevention of chronic systemic inflammation in high-fat diet-induced obesity by exercise training [8]. Physical exercise protects asthmatic children against increased oxidative stress induced by their respiratory system malfunction [19].

One important mechanism for beneficial effects of exercise is upregulation of antioxidant and anti-inflammatory defense systems in various tissues. It has been reported that lung, heart and liver antioxidant and anti-inflammatory enzymes activities increased significantly in rats which performed exercise compared to the control sedentary rats [4]. This presumably is due to mildly increased levels of oxidative stress that occurs during exercise. Low amounts of ROS produced during regular skeletal muscle work are a part of “hormesis”, which describes the generally favorable biological responses to low exposures to stressors [3].

Paradoxically, exercise can induce oxidative damages. Huang et al. have reported that physical exercise induced oxidative stress which could cause damage to muscle and liver tissues [6]. Dalla Corte et al. have demonstrated elevation of oxidative damage markers in the brain, skeletal muscle, and blood after exhaustive exercise [20]. Such deleterious effects are due to duration and intensity of the exercise. When animals undergo exhaustive physical activity, ROS overproduction causes oxidative damages. So, intensity and duration are determinant factors to be considered in the exercise experiments. In the present study, we prepared voluntary exercise facility for the animals that is regarded as mild/moderate intensity of physical activity [8]. 

Lead is a heavy metal which is used in more than 900 industries [9]. The phasing out of leaded gasoline for transportation and the removal of lead from paint has resulted in substantial lowering of mean blood lead levels. However, because lead is a persistent metal, it is still present everywhere in the environment—in water, soil, and imported products manufactured with lead [21]. Exposure to lead produces various deleterious effects on the hematopoietic, renal and central nervous system, mainly through increased oxidative stress. Different antioxidants have been successfully used in previous studies to prevent or treat lead toxicity, including vitamins, flavonoids, alpha lipoic acid, and herbal antioxidants like garlic [22]. Our results indicated that mild exercise as a known antioxidant systems enhancer has protective effects against lead toxicity as well. 

## 5. Conclusion

The results of the present study demonstrated that voluntary exercise prevented elevation of lead-induced oxidative stress and inflammation markers in rat blood. Therefore, chronic mild exercise might be effective in prevention of oxidative stress and inflammation induced by lead toxicity. Finally, we suggest that voluntary exercise increases body resistance against lead-induced oxidative stress and inflammation.
